# Urinary tract infections decreased in Finnish children during the COVID-19 pandemic

**DOI:** 10.1007/s00431-022-04389-9

**Published:** 2022-01-31

**Authors:** Ilari Kuitunen, Miia Artama, Marjut Haapanen, Marjo Renko

**Affiliations:** 1 Institute of Clinical Medicine, Department of Pediatrics, University of Eastern Finland, Kuopio, Finland; 2 Department of Pediatrics, Mikkeli Central Hospital, Porrassalmenkatu 35-37, 50100 Mikkeli, Finland; 3 Faculty of Social Sciences, Department of Epidemiology, Tampere University, Tampere, Finland; 4 Finnish Institute of Health and Welfare, Tampere, Finland; 5 Department of Pediatrics, Kuopio University Hospital, Kuopio, Finland; 6 PEDEGO Research Unit, University of Oulu, Oulu, Finland

**Keywords:** COVID-19, Cystitis, Pyelonephritis, Epidemiology, Non-pharmaceutical interventions

## Abstract

Social restrictions reduced the rates of respiratory infections in 2020, but studies on the rates of urinary tract infections (UTIs) during lockdown have had conflicting results. This study aimed to report UTI incidence during the first and second waves of COVID-19 pandemic in Finland. We conducted a retrospective register-based cohort study. The whole Finnish pediatric population (children under the age of 15 years, *N* = 860,000) was included. The yearly and monthly incidences of UTIs per 100,000 children in 2020 were compared to that of three previous years (2017–2019) by incidence rate ratios (IRRs) with 95% confidence intervals (CIs). A total of 10,757 cystitis and 4873 pyelonephritis cases were included. The yearly incidence of cystitis was 12% lower (IRR 0.88, CI 0.83–0.94) among children aged 1–6 in 2020 and 11% (IRR 0.89, CI 0.83–0.95) lower among children aged 7–14 in 2020 compared with previous years. The yearly incidence of pyelonephritis was 16% lower (IRR 0.84, CI 0.76–0.94) among children aged 1–6. No significant decrease were observed among children aged < 1 and 7–14.

*Conclusion*: The incidence of cystitis and pyelonephritis during a period of social restrictions was lower than during 2017–2019, especially in children aged 1–6 years. These results raise the possibility of reducing the occurrence of urinary tract infections in children by improving hygiene measures.

**Table:** 

**What is known:** *• Social restrictions have reduced the rate of common respiratory infections globally.* *• Previous studies have presented a decreased or unchanged incidence of urinary tract infections during the COVID-19 pandemic.*	
**What is new:** *• During the pandemic, there was a decrease in the incidence of urinary tract infections in Finnish children and the most prominent decrease was in daycare-aged children.* *• Improved hygiene measures and social restrictions may have influenced the transmission of uropathogens.*

**Supplementary Information:**

The online version contains supplementary material available at 10.1007/s00431-022-04389-9.

## Introduction

When the World Health Organization (WHO) declared severe acute respiratory corona virus 2 (SARS-CoV-2) a pandemic in March 2020, a majority of countries implemented social restrictions and lockdowns to prevent the spread of COVID-19. In Finland, schools and daycares were closed from March to May 2020. Previous studies have shown that these restrictions had a substantial impact on globally decreasing the incidence of common respiratory infections [1–4], acute otitis media cases [1, 5–7], and overall pediatric emergency department visit rates [8–13]. Finnish schools reopened in May 2020 and have remained open since. The reopening of schools had no effect on the spread of respiratory infections in Finland [14]. During the second wave, the strategy to limit the spread of COVID-19 involved minimal restrictions imposed on children. These restrictions were effective in preventing the spreading of respiratory syncytial virus [15] and influenza in fall 2020 [16], but the overall rate of visits to pediatric emergency departments has been normal since summer 2020 [17].

A previous study from the US found that the visit rate due to urinary tract infections (UTI) decreased during the period of lockdown and social restrictions [5]. Also, a recent review found that the rate of non-communicable infectious diseases decreased slightly in March 2020 when the lockdowns began [13]. A few additional reports have shown that the rates of UTI remained stable despite the social restrictions [7, 10]. Interestingly, a study in Italy found that the rate of UTIs increased in tertiary level units during the lockdown period [12].

The current understanding of pathomechanisms for UTI is the ascending route, the hematogenous route, and the lymphatic route [18]. Especially in young children, anomalies and obstructions causing urinary stasis might increase the likelihood of UTI [19]. In recent years, the pathogenesis and mechanisms of UTI in children have gained more interest, and alternative theories have been studied and presented [20]. It is possible that improved hygiene measures could reduce the transmission of uropathogens from person-to-person or from gut to urinary tract via hands and, therefore, decrease rates of UTI, although UTI is classically defined as a non-communicable disease.

We aimed to evaluate the incidence of urinary tract infections in Finnish children in 2020 during the first and second waves of the COVID-19 pandemic and compare it to that of previous years.

## Methods

Data for this retrospective, nationwide, register-based cohort study were gathered from the national open-access care registers in Finland. The National Care Register is maintained by the Finnish Institute of Health and Welfare, and it includes all visits and hospitalizations in specialized health care [21]. Specialized health care in Finland means public secondary and tertiary level pediatric hospitals, for which all Finnish inhabitants are eligible without insurance. The Primary Health Care register is also maintained by the Finnish Institute of Health and Welfare, and it includes all visits for public primary care, which is publicly funded and open to anyone [22]. Private sector health care is not included in the current study. The whole Finnish pediatric population of 860,000 children (in 2020) was included in this register-based study.

For this report, we gathered age-stratified monthly numbers of visits due to UTI (cystitis and pyelonephritis) in secondary/tertiary health care for children aged < 1 year, 1–6 years, and 7–14 years of age from January 2017 to December 2020. UTIs were classified based on the ICD-10 classification. From the primary care register, the yearly visit numbers for children aged 1–6 and 7–14 were gathered for cystitis. Cystitis included diagnosis codes N30.0 and N30.9. Pyelonephritis included diagnosis code N10.

The first wave of COVID-19 lasted from March to May, and the second wave began in September 2020. The restrictions implemented in Finland during 2020 were as follows:

- From January 1 to March 16, there were no restrictions.
- From March 16 to May 16, schools and daycares were closed, gatherings were limited, and a lockdown without curfew was in force.
- From May 16 to September, minimal restrictions were imposed on children, there were no restrictions on outdoor gatherings, and schools and daycares were fully open. Travelling was enabled to low-incidence countries during July and August.
- From September to December, instead of nationwide restrictions, regional stepwise restrictions based on the 14-day cumulative incidence of COVID-19 cases in specific regions were implemented. Schools and daycares remained open. Travel was restricted.

The monthly and yearly incidence with 95% confidence intervals (CI) per 100,000 children in each age group was calculated by the Poisson exact method, and the incidence rate in 2020 was compared to the mean incidence during 2017–2019 by incidence rate ratios (IRRs) with CI. The age-stratified population was gathered for each year from the open-access population reports of Statistics Finland [23]. The number of children living in Finland at the end of each year was used as the nominator in the incidence calculations. This study has been reported according to the EQUATOR guideline of REporting of studies Conducted using Observational Routinely-collected health Data (RECORD).

Due to the retrospective register-based design, ethical committee evaluation was not required or obtained.

## Results

### Cystitis

**Fig. 1:**
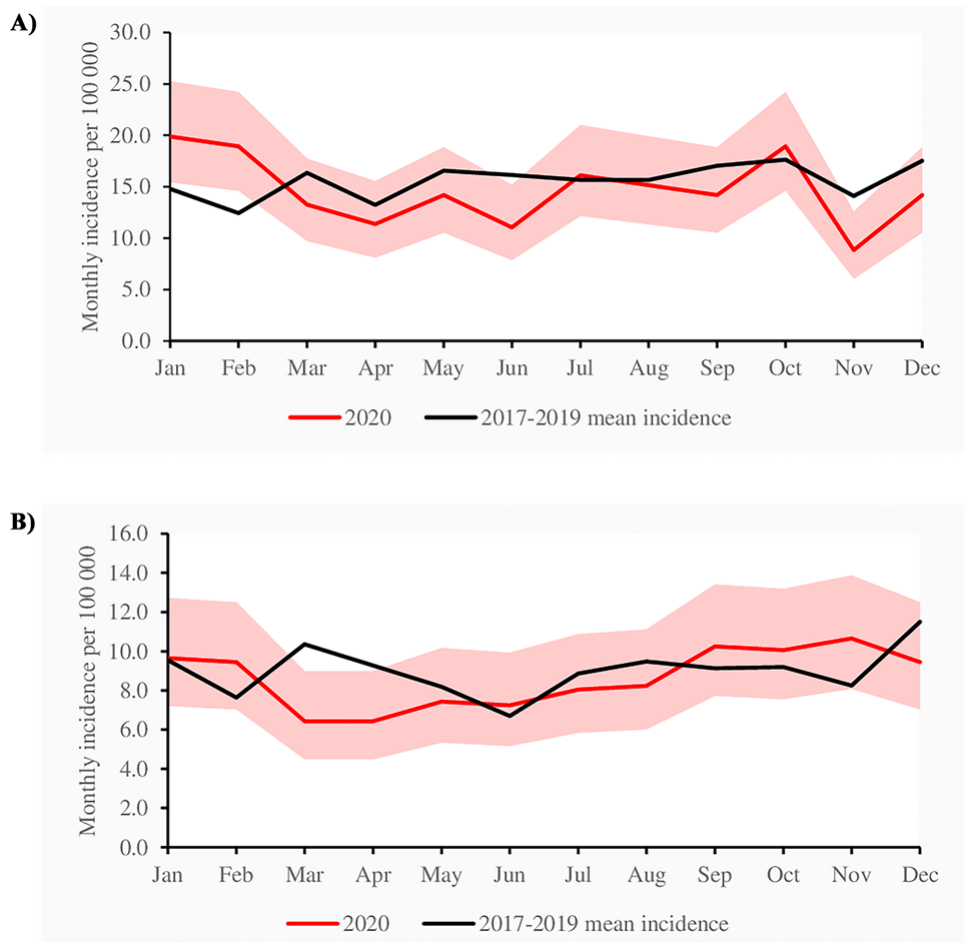
**A** Monthly incidence of cystitis per 100,000 children aged 1–6 diagnosed treated in specialized healthcare in Finland. Light pink area indicates 95% confidence intervals. **B** Monthly incidence of cystitis per 100 000 children aged 7–14 diagnosed and treated in specialized healthcare in Finland. Light pink area indicates 95% confidence intervals

A total of 10,757 cystitis cases were included and, of these, 2389 (22.2%) were treated in 2020. The incidence in children aged 1–6 years was 394 per 100,000 in 2020 and 433 per 100,000 in 2017–2019 (IRR 0.88, CI 0.83–0.94; Table 1). The corresponding incidence in children aged 7–14 was 229 in 2020 and 250 in 2017–2019 (IRR 0.89, CI 0.83–0.95: Table 1). The IRR in children aged 1–14 for diagnose in primary care was 0.83 (CI 0.78–0.88) and in specialized care 0.94 (0.88–1.00: Table 1). Yearly numbers and incidences of cystitis are presented in Supplementary Table 1. During the lockdown in March 2020, the incidence dropped below the reference level in both age groups and returned to near normal levels during the summer (Fig. 1). A second drop was seen among children aged 1–6 in November 2020 (IRR 0.63, CI 0.42–0.95: Fig. 1A).

**Table 1:** The yearly incidence of cystitis per 100,000 children in Finland

	2017–2019				2020						
Age	*N*	Incidence	95 CI		*N*	Incidence	95 CI		IRR	95 CI	
			Lower	Upper			Lower	Upper		Lower	Upper
Secondary/tertiary care											
1–6 years	1911	187.1	178.9	195.6	558	176.1	162.0	191.2	0.94	0.86	1.03
7–14 years	1598	108.1	102.8	113.4	514	103.3	94.7	112.9	0.96	0.87	1.06
1–14 years	3509	140.4	135.8	145.1	1072	131.6	123.9	139–7	0.94	0.88	1.00
Primary care											
1–6 years	2639	258.4	248.7	268,4	691	218.1	202,3	234.8	0.84	0.78	0.92
7–14 years	2220	150.2	143.9	156,4	626	125.8	116.3	136.5	0.84	0.77	0.92
1–14 years	4859	194.4	189.0	199.9	1317	161.7	153.2	170.6	0.83	0.78	0.88
Combined											
1–6 years	4550	445.5	432.7	458,6	1249	394.2	372.8	416.5	0.88	0.83	0.94
7–14 years	3818	258.3	249.9	266,3	1140	229.1	216.1	243.6	0.89	0.83	0.95
1–14 years	8386	334.8	327.6	342.0	2389	293.4	281.8	305.3	0.88	0.84	0.92

### Pyelonephritis

**Fig. 2:**
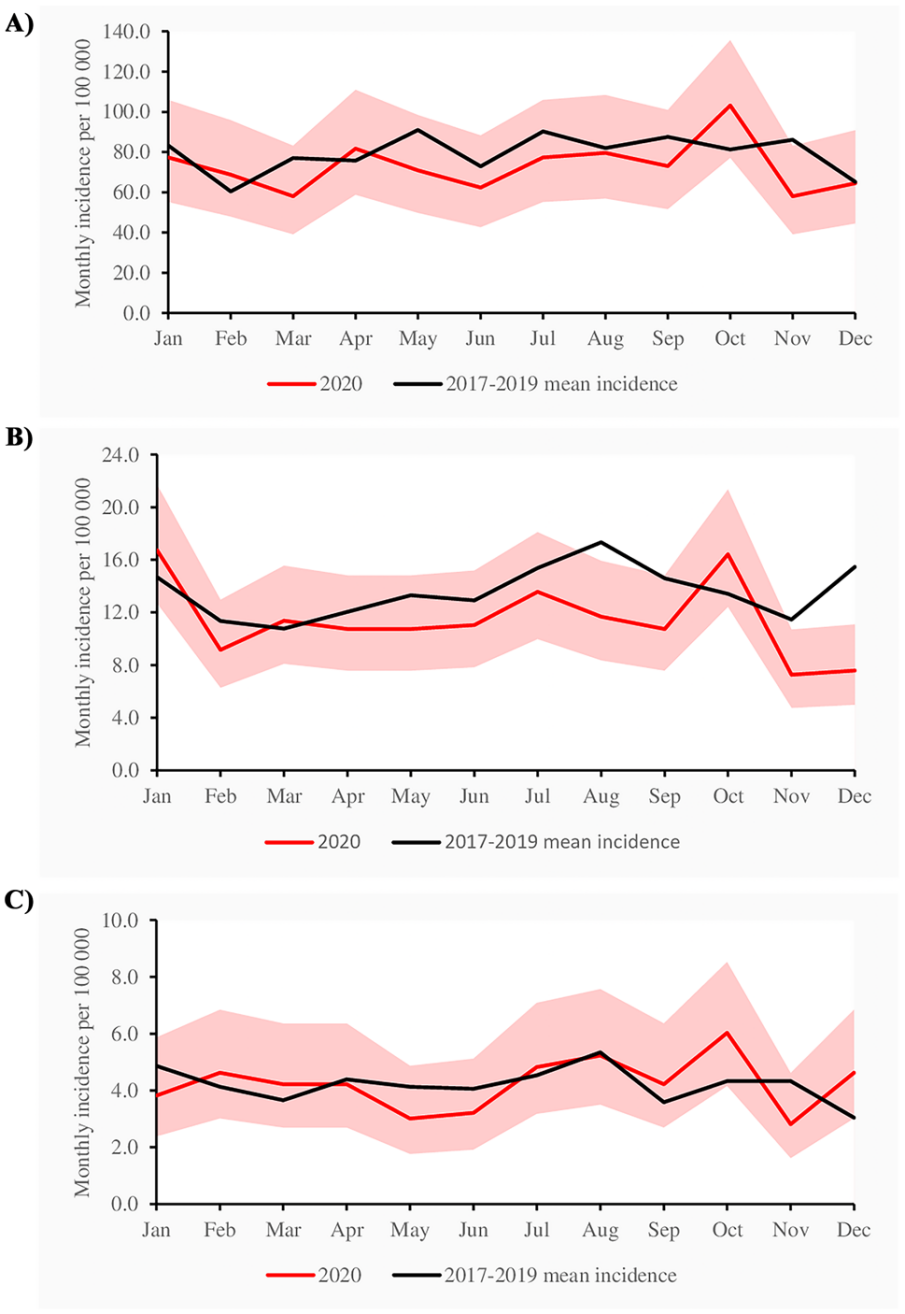
**A** Monthly incidence of pyelonephritis per 100,000 children aged less than 1 year diagnosed and treated in specialized healthcare in Finland. Light pink area indicates 95% confidence intervals. **B** Monthly incidence of pyelonephritis per 100,000 children aged 1–6 diagnosed and treated in specialized healthcare in Finland. Light pink area indicates 95% confidence intervals. **C** Monthly incidence of pyelonephritis per 100,000 children aged 7–14 diagnosed and treated in specialized healthcare in Finland. Light pink area indicates 95% confidence intervals

A total of 4873 pyelonephritis cases were included and, of these, 1094 (22.5%) were treated in 2020. The overall incidence for pyelonephritis was 127 per 100,000 children in 2020 and 143 per 100,000 children aged 0–14 in 2017–2019 (IRR 0.89, CI 0.83–0.95: Table 2). Yearly numbers and incidences of pyelonephritis are presented in Supplementary Table 2. The decrease was mostly seen among children aged 1–6 years, in whom the incidence in 2020 was 16% lower than in the reference years (IRR 0.84, CI 0.76–0.94). The monthly incidence remained near to reference levels among the youngest age group (Fig. 2A). Among children aged 1–6, the incidence was lower from April to September and again in November (Fig. 2B). A greater variation was observed among children aged 7–14 in the monthly incidence in 2020 (Fig. 2C).

**Table 2:** The yearly incidence of pyelonephritis per 100,000 children in Finland in specialized healthcare

	2017–2019				2020						
Age	*N*	Incidence	95 CI		*N*	Incidence	95 CI		IRR	95 CI	
			Lower	Upper			Lower	Upper		Lower	Upper
< 1 year	1372	952.8	903.4	1004.2	407	874.8	792.9	963.0	0.92	0.82	1.03
1–6 years	1662	162.7	155.1	170.7	434	137.0	124.5	150.3	0.84	0.76	0.94
7–14 years	745	50.4	46.8	54.1	253	50.9	44.9	57.6	1.01	0.88	1.16
0–14 years	3779	142.9	138.4	147.6	1094	127.1	119.7	134.8	0.89	0.83	0.95

## Discussion

Social restrictions seemed to decrease the incidence of UTIs during the COVID-19 pandemic, especially among children aged 1–6 years. The decrease was seen in both cystitis and pyelonephritis cases. In Finland, this age group attends daycare and typically carries a heavy infectious load, especially of respiratory and gastrointestinal infections.

**Fig. 3:**
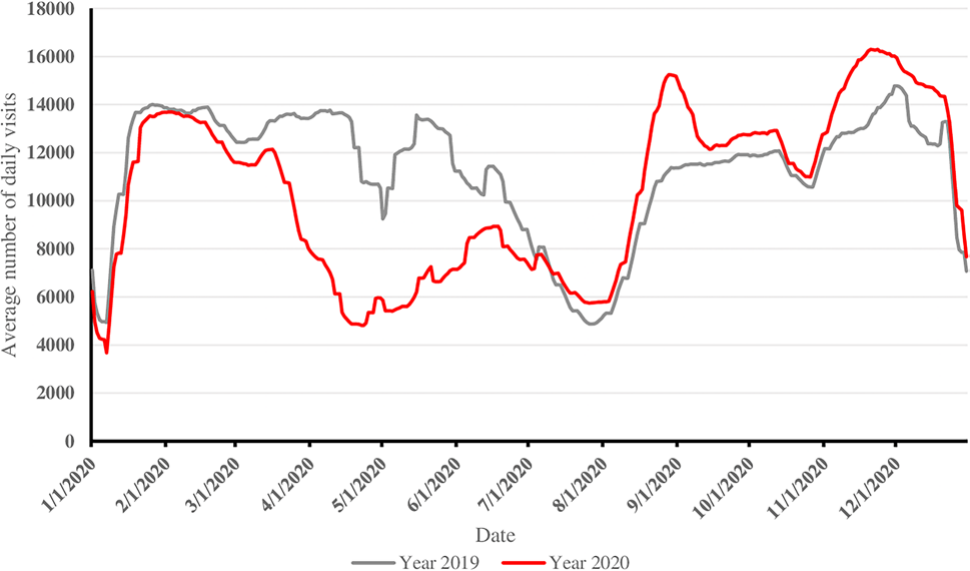
Nationwide 14-day rolling mean daily visit rate for children aged 0–14 years in primary health care from 2019 to 2020 in Finland

Our results are in line with a study from the US in which lower numbers of UTIs were detected during and right after the lockdown [5]. The studies that have reported no difference in UTI rates have been smaller and, therefore, at higher risk for being underpowered to detect changes [7, 10]. We present the first nationwide results of urinary tract infections during this pandemic and restriction period. Interestingly, the Italian results from a tertiary level pediatric hospital indicated that pyelonephritis cases increased during the lockdown [12]. This might have been due to patient selection as the availability of primary care resources was impacted during the major lockdown. Also, in Italy, during the first wave, the incidence of SARS-CoV-2 was high, and patients might have waited longer to seek treatment, so UTIs might have had time to evolve into pyelonephritis. Finland has had the lowest rates of COVID-19 cases in Europe, yet primary care visit rates were lower in March and April 2020 than in previous years. However, by May 2020, the visit rates had normalized and, since then, have remained above the reference level (Fig. 3). A recent report from Sweden showed similarly that the rate of UTIs increased slightly during the pandemic, although all other infections decreased [25]. This is an interesting finding as the strategy in Sweden has differed greatly from that of other European countries. The restrictions implemented in Sweden have been minimal and not as strict as here in Finland. Therefore, it could be speculated that stricter restrictions might have influenced the dynamics of UTI transmission in Finland.

UTIs have been classically considered to be non-communicable, and the infection is thought to arise from the patient’s own bacterial flora. However, our results suggest that during the social restrictions, with improved hand hygiene guidance and stay-at-home orders, the incidence of urinary tract infection was lower. This was a surprising finding. Previous findings of studies on the similar uropathogenic *Escherichia coli* strains in sexual partners suggest that UTIs could be transmitted during intercourse [26, 27]. In addition to these studies, we were unable to find previous reports discussing the potential person-to-person spreading. Our results raise a question about whether the pathomechanisms and transmission dynamics of UTIs should be re-examined.

One possible explanation for our results could be that people preferred tele-health visits during the pandemic, but in Finland, the Current Care guidelines advice that UTIs in children be diagnosed and treated only after a urine sample is taken. To avoid treating of asymptomatic bacteriuria, the guidelines also instruct that urine samples should only be taken from children presenting symptoms suitable for UTI [24]. Due to the nature of our data, we do not know for sure if UTI diagnoses during 2020 were made in a manner similar to that in previous years. Furthermore, it might be possible that the diagnostic process is more specific in specialized healthcare than in primary care due to the better capacity of personnel and laboratory services. In our results, clearer decrease in cystitis incidence was observed in primary care than in secondary care, but the decrease in secondary care was borderline significant. Diagnostic accuracy of UTIs in primary care setting compared to specialized healthcare in children has not been studied in Finland.

The main strength of our study is the nationwide coverage with the register-based approach. The care register of specialized health care has a high validity, and approximately 95% of the visits are reported there [21]. The primary care register has had some problems with reporting quality, but it has improved over time and, during our study period, the reporting quality remained unchanged as over 90% of the publicly funded health care centers provided information to the register [22]. The main limitation of our study is the lack of data on hospital admissions, which could have helped to estimate the severity of diagnosed pyelonephritis cases. Further limitation is the lack of testing numbers as these are not reported to the registers. Therefore, we are unable to use test-negativity design to analyze the impact of reduced primary care resources and possible reduced urine testing numbers on the rates of UTI. However, it must be noted that as urine samples are only taken from symptomatic children, the decrease in UTI incidence would lead to lower rates of samples tested.

During the social restrictions, reduced incidences of pediatric cystitis and pyelonephritis were observed, especially in children aged 1–6 years. These results raise a question about whether the rates of urinary tract infections could be reduced by improving hygiene measures in daycare-aged children.

## Supplementary Information

Below is the link to the electronic supplementary material.

Supplementary file1 (DOCX 13 KB)

## Data Availability

Available upon request from the corresponding author.

## Abbreviations

CI: Confidence interval
ED: Emergency department
IRR: Incidence rate ratio
UTI: Urinary tract infection
