# Supplementary material for: Urinary tract infections decreased in Finnish children during the COVID-19 pandemic
Source: Eur J Pediatr. 2022 Jan 31;181(5):1979–84. doi: 10.1007/s00431-022-04389-9 (PMC8801286; doi:10.1007/s00431-022-04389-9)
Supplement: Supplementary file 1 — Supplementary file1 (DOCX 13 KB) [file 431_2022_4389_MOESM1_ESM.docx]

Supplementary table 1: Yearly numbers and incidences of cystitis cases treated in primary care and specialized healthcare for patients aged 1 to 14 years from 2017 to 2020.

|  |  |  | 95% CI | |
| --- | --- | --- | --- | --- |
| Year | N | Incidence | Lower | Upper |
| 2017 | 2517 | 300 | 288 | 312 |
| 2018 | 2968 | 356 | 343 | 369 |
| 2019 | 2892 | 350 | 338 | 363 |
| 2020 | 2389 | 293 | 282 | 305 |
|  |  |  |  |  |

Supplementary table 2: Yearly numbers and incidences of pyelonephritis cases treated in specialized healthcare for patients aged 0 to 14 years from 2017 to 2020.

|  |  |  | 95% CI | |
| --- | --- | --- | --- | --- |
| Year | N | Incidence | Lower | Upper |

| 2017 | 1234 | 139 | 131 | 147 |
| --- | --- | --- | --- | --- |
| 2018 | 1253 | 142 | 134 | 150 |
| 2019 | 1292 | 148 | 140 | 157 |
| 2020 | 1094 | 127 | 120 | 135 |
